# Relationships between Oxidative Stress, Liver, and Erythrocyte Injury, Trace Elements and Parasite Burden in Sheep Naturally Infected with *Dicrocoelium dendriticum*

**Published:** 2017

**Authors:** Hadi SAMADIEH, Gholam-Reza MOHAMMADI, Mohsen MALEKI, Hassan BORJI, Mohammad AZIZZADEH, Mohammad HEIDARPOUR

**Affiliations:** 1. Dept. of Clinical Sciences, School of Veterinary Medicine, Ferdowsi University of Mashhad, Mashhad, Iran; 2. Dept. of Pathobiology, School of Veterinary Medicine, Ferdowsi University of Mashhad, Mashhad, Iran

**Keywords:** Ovine dicrocoeliasis, Oxidative stress, Parasite burden

## Abstract

**Background:**

The aim of this study was to investigate the role of oxidative stress in the pathology of ovine dicrocoeliasis.

**Methods:**

During Dec 2013 – Oct 2014, seventy-two sheep (1–3 years) with liver dicrocoeliasis along with 47 healthy sheep were selected from animals admitted for slaughtering at slaughterhouse located in Neyshabour, Razavi Khorasan Province, Iran.

**Results:**

In comparison to healthy control, the malondialdehyde (MDA) level and serum total antioxidant capacity were significantly higher in the parasitized group (*P*<0.05). A significant increase in liver MDA concentration (*P*<0.05) of parasitized group was also observed. Packed cell volume (PCV), zinc, iron, total bilirubin and albumin sera levels were significantly lower in the parasitized group (*P*<0.05). In parasitized sheep, a significant positive correlation was seen between serum MDA concentration and the activity of aspartate aminotransferase (AST). On the other hand, the concentration of serum MDA was inversely correlated with the value of PCV. No significant differences were observed for MDA concentration and total antioxidant capacity between normal and abnormal hepatic lobes in the parasitized animals. Oxidative stress markers (MDA and total antioxidant capacity in serum and liver samples) showed no significant correlations with the extent of pathological lesions and serum variables of liver injury in the parasitized sheep. No significant correlation was observed between oxidative stress markers and the fluke’s number in the parasitized animals.

**Conclusion:**

Oxidative stress may play an important role in the erythrocyte destruction in sheep naturally infected with *D. dendriticum.* However, no clear relationships were observed between the oxidative stress, hepatic damage and parasite burden.

## Introduction

Dicrocoeliasis is a parasitic disease caused by *Dicrocoelium dendriticum.* This parasite lives in the bile ducts and gall bladder of ruminants as well as many other animal species including human. *D. dendriticum* has a worldwide distribution and is more prevalent throughout Europe, Asia, North Africa and North America (1). Dicrocoeliasis is one of the most common parasitic diseases in small ruminants, and the susceptibility of sheep to dicrocoeliasis is more than goats (2). The clinical symptoms of dicrocoeliasis are mild in most affected animals. However, liver function impairment and subsequent reduces in milk and meat production cause severe economic losses. “The young flukes migrate directly up the biliary duct system of the liver without penetrating the gut wall, liver capsule or liver parenchyma” (2). Pathological injuries in the liver and gall bladder are probably caused by the toxic metabolites of parasite and the mechanical stimulation of the walls of bile ducts by the fluke (1). There was a direct relationship between parasite number and lesion scores in animals with dicrocoeliasis (3). Increased hepatic enzyme activities, aspartate aminotransferase (AST) and alanine aminotransferase (ALT), were observed in lambs experimentally infected with 1000 and 3000 metacercariae of *D. dendriticum*. No significant correlation was observed between the increased hepatic enzyme activities and the parasite density, although the highest activities were revealed in the lambs with the greatest parasite numbers (4).

Host immune systems protect the animal from parasites via cellular mechanism (5). Increased generation of reactive oxygen species (ROS) is the first reaction of immune cells (6). Defense mechanisms associated with the production of ROS are very important in the resistance of host to infection by the liver fluke (7). Although ROS have an important role in defense against the invading parasite, but when produced in high amounts, they can result in oxidative damage to host tissues (8). In addition, bile acids are strong pro-oxidants that their accumulation in biliary ducts could result in tissue damage mediated by ROS production (9). To counteract the toxic action of ROS, host cells have several antioxidant mechanisms that include antioxidant enzymes and nutritional antioxidants (10). In addition, trace elements such as zinc, copper, and iron, are essential components of certain endogenous antioxidants. For example, copper and zinc are required for the activity of superoxide dismutase (11).

Oxidative stress is an imbalance between the generation of ROS and the ability of antioxidants to neutralize their harmful effects. The presence of oxidative stress have been reported in several parasitic infections including the infections with *D. dendriticum* in sheep (12) and hamsters (13), *Fasciola hepatica* in sheep (14) and rats (7), and *Echinococcus granulosus* in cattle (15), camel (16) and sheep (17). Although oxidative liver damage caused by *D. dendriticum* has been described in experimentally infected hamsters (13,18), the role of oxidative stress in the liver and erythrocyte injury, as well as the correlations between parasite burden and oxidative stress markers and trace elements changes have not been evaluated in natural ovine dicrocoeliasis.

The objectives of the present study were to 1) investigate the relationship of oxidative stress markers [malondialdehyde (MDA) and total antioxidant capacity] with serum variables of liver injury [AST, gamma glutamyl transferase (GGT), bilirubin, albumin], extent of liver injury (based on histopathological examination) and anemia (PCV value) in sheep with dicrocoeliasis; 2) evaluate the relationship of oxidative stress markers with parasite burden of *D. dendriticum* in the liver; and 3) to compare trace elements in parasitized and healthy animals.

## Materials and Methods

### Animals

During Dec 2013 – Oct 2014, 72 sheep (1–3 yrs.) with liver dicrocoeliasis along with 47 healthy sheep were selected from animals admitted for slaughtering at slaughterhouse located in Neyshabour, Razavi Khorasan Province, Northeastern Iran. The selection of parasitized animals was restricted to those affected with liver dicrocoeliasis only. In addition, the negative control animals did not show any pathology and parasite in the carcass and blood samples. The infection status of the selected animals was also confirmed by parasitological method in the laboratory as described in parasitological examination.

### Sampling

From each sheep, two blood samples were collected by the jugular venipuncture, one in a tube containing ethylenediaminetetraacetic acid dipotassium salt (EDTA-K_2_) and the second in the tube without the anticoagulant for subsequent serum collection. The blood samples anticoagulated with EDTA were used for parasitological examination and packed cell volume (PCV) determination. The serum was separated by centrifugation at 1800×g for 10 min and stored at −20°C until analysis. For parasitological and histopathological examination and measurement of oxidative stress markers in the hepatic tissue, liver was also collected from each sheep.

The experiment was approved by the Animal Welfare Committee of the School of the Veterinary Medicine, Ferdowsi University of Mashhad, Mashhad, Iran.

### Hematological and parasitological examination

PCV concentration was determined by microhaematocrit method (19). The blood samples with anticoagulant were also used to prepare thin blood smears for parasitological examination. Blood smears were stained with giemsa for 30 min and then examined for the presence of blood parasites under light microscopy. The blood smears were recorded as negative if no parasites were observed in 200 oil-immersion fields (1000×).

In the laboratory, the livers and gall bladders were subjected to thorough investigation for the collection of parasites and parasitic materials. The techniques used for the recovery and counting of *D. dendriticum* from the liver are as described (20). The animals with liver dicrocoeliosis and no other pathology and parasite in the carcass, liver, gall bladder and blood samples were selected as parasitized group. The negative control animals did not show any pathology and parasite including *D. dendriticum* in their samples.

### Biochemical analysis

Serum concentration of bilirubin, albumin, zinc, copper and iron and serum activity of AST and GGT were measured by commercial kits [Pars Azmoon, Iran for iron, AST, GGT, bilirubin and albumin; Giesse Diagnostics, Italy for zinc; EliTech diagnostics, France for copper] using an autoanalyser (Biotecnica, Targa 3000, Rome, Italy).

### Oxidative stress markers

Oxidative stress markers were determined in the serum and in the liver samples collected from the visceral surface of the liver: left lobe, right lobe, caudate lobe, quadrate lobe. For determination of oxidative stress markers in liver, the tissue samples obtained from different lobes were minced, cut into small pieces and then dried on a filter paper and homogenized (10% w/v) in ice-cold 1.15% KCl-0.01 M sodium, potassium phosphate buffer (pH 7.4) by “Silent crusher M” type homogenizer (Heidolph Instruments GmbH & Co. KG, Schwabach, Germany). The homogenate was centrifuged at 18000g for 20 min at 4 °C, and the resultant supernatant was used for the determination of oxidative stress markers.

The concentration of MDA, as a marker of lipid peroxidation, in liver and serum samples was determined as thiobarbituric acid reactive substances (21). The method is dependent on forming a color complex between MDA and thiobarbituric acid (TBA). Briefly, 0.2 ml of serum or homogenized tissues was added to 1.3 ml of 0.2 mol/l tris, 0.16 mol/l KCl buffer (pH 7.4). TBA (1.5 ml) was added and the mixture was heated in a boiling water bath for 10 min. After cooling, 3 ml of pyridine– butanol (3:1, v/v) and 1 ml of 1 mol/l NaOH were added. The absorbance was read at 548 nm against bidistilled water as a blank. The nmol of MDA per milliliter of serum or homogenized tissues was calculated using 1.56×10^5^ as extinction coefficient.

The total antioxidant capacity of the homogenized tissues and serum samples was measured using ferric reducing antioxidant power (FRAP) assay, which depends upon the reduction of ferric tripyridyl triazine (Fe(III)-TPTZ) complex to the ferrous tripyridyl triazine (Fe(II)-TPTZ) by a reductant at low pH. Fe (II)-TPTZ has an intensive blue color and can be monitored at 593 nm (22). The FRAP values were determined by extrapolation from the standard curve and were expressed in nanomoles per milliliter.

### Histopathology

Histopathological samples of infected and negative control animals were collected from different hepatic lobes (from the same locations collected for oxidative stress markers). Pieces of the liver tissue about one cubic cm. in size were fixed in 10 percent buggered neutral formalin. All the sections were stained routinely with Haematoxylin and Eosin for detailed histopathological examinations.

### Statistical analysis

Controlling for age, sex and existence of other liver infestation, linear regression model was used to evaluate the effects of dicrocoeliasis on PCV, serum copper, zinc, iron, total bilirubin, albumin, GGT and AST levels and liver and serum oxidative stress markers. The relationship between oxidative stress markers with extent of liver injury, parasite burden, hematological and biochemical variables was performed by Pearson and Spearman’s correlation test. Comparison of oxidative stress markers between normal hepatic lobes (hepatic lobes with no parasite and pathological lesion) and abnormal hepatic lobes (hepatic lobes with *D. dendriticum* and pathological lesions) in parasitized animal was performed by Mann-Whitney U test. All analyses were carried out using SPSS software ver. 21 (Chicago, IL, USA) and *P*<0.05 considered as significant.

## Results

### Hematological and biochemical findings

The results of haematological analysis (Table 1) indicated that the level of PCV was significantly *(P*<0.05) lower in sheep with dicrocoeliasis than those of healthy controls.

**Table 1:** Trace elements, hepatic markers and PCV levels in sheep with dicrocoeliosis and healthy Controls

**Parameter**	**Parasitized**	**Healthy**	***P*-value**
PCV (%)	22.31 ± 8.42	38.35 ± 5.82	*P*<0.01
Zinc (μg/dL)	162.17 ± 34.02	179.52 ± 29.59	*P*<0.01
Copper (μg/dL)	130.79 ± 106.06	109.32 ± 34.75	*P*=0.17
Iron (μg/dL)	115.68 ± 22.72	134.42 ± 32.55	*P*<0.001
Fibrinogen (mg/dL)	330.98 ± 153.61	389.79 ± 179.40	*P*=0.057
Total protein (g/dL)	7.12 ± 0.86	7.31 ± 0.65	*P*=0.21
Albumin (g/dL)	3.79 ± 0.64	4.34 ± 0.37	*P*<0.001
Total Bilirubin (mg/dL)	0.58 ± 0.04	0.71 ± 0.08	*P*<0.001
AST(U/L)	148.81± 52.84	131.85 ± 33.99	*P*=0.053
GGT(U/L)	51.28 ± 18.71	56.00 ± 13.53	*P*=0.150

The zinc, iron, total bilirubin, and albumin sera levels were found to be significantly lower in the parasitized sheep compared to the healthy sheep (*P*<0.001, Table 1). No significant differences were observed for serum copper, GGT, AST and total protein between parasitized and healthy groups (Table 1).

### Histopathological findings

**Fig. 1:**
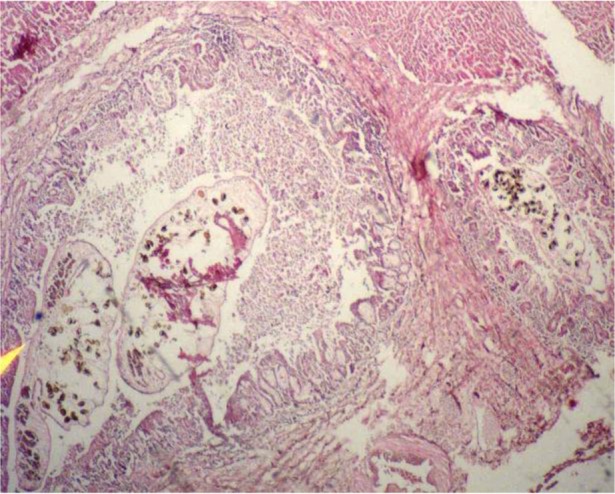
Histopathological appearance of a bile duct with adult worm and eggs of *D. dendriticum*; superficial erosive effect of the parasite sucker on the lining of epithelial cells, leukocytic infiltration and periductal fibrosis. Original picture from a parasitized sheep stained with Haematoxylin and Eosin (HE), 4×

Pathological changes in the biliary system demonstrated different degree of hyperplasia, desquamation, and necrosis of mucosal epithelium. The lumen of these bile ducts often included some worms and a superficial erosive effect of the parasite sucker was seen on the lining of epithelial cells (Fig. 1). Leukocytic infiltration (macrophages, eosinophils, lymphocytes and plasma cells) and periductal fibrosis were also observed. Occasional ectopic worms were detected inside parenchyma that forms foreign body granulomatous inflammation in liver.

### Oxidative stress markers

The levels of serum MDA and ferric reducing antioxidant power assay (FRAP) were significantly (*P*<0.05) higher in sheep with dicrocoeliasis than those of healthy controls (Table 2). To compare the oxidative stress markers in liver of parasitized and healthy groups, MDA and FRAP were measured in different hepatic lobes of each animal and the average value was calculated. A significant increase in liver MDA concentration (*P*<0.05) of parasitized group was observed when compared to healthy group (Table 2). No significant difference was seen in FRAP level of liver between parasitized and healthy sheep (Table 2).

**Table 2:** Oxidative stress markers in blood serum and liver of sheep with dicrocoeliosis and healthy controls

**Parameter**	**Parasitized**	**Healthy**	***P*-value**
Liver MDA (nmol/ml)	2.63 ± 6.97	2.85 ± 1.585	*P*<0.001
Serum MDA (nmol/ml)	23.64 ± 8.90	12.89 ± 4.09	*P*<0.001
Liver FRAP (mmol/L)	0.165 ± 0.051	0.163 ± 0.047	*P*=0.818
Serum FRAP (mmol/L)	0.498 ± 0.140	0.397 ± 0.080	*P*<0.001

In addition, the oxidative stress markers were compared between normal hepatic lobes (hepatic lobes with no parasite and pathological lesion) and abnormal hepatic lobes (hepatic lobes with *D. dendriticum* and pathological lesions) in each parasitized animal. No significant differences were observed for MDA and FRAP between normal and abnormal lobes in parasitized animals (Table 3).

**Table 3:** Oxidative stress markers in normal and abnormal hepatic lobes in parasitized sheep

	**Abnormal**			**Normal**			***P*-value**
	Q1	Q2 (Median)	Q3	Q1	Q2 (Median)	Q3	
MDA (nmol/ml)	4.01	7.23	9.94	4.06	6.09	9.94	0.688
FRAP (mmol/L)	0.10	0.16	0.22	0.12	0.16	0.21	0.812

Q1, Q2, and Q3 (quartiles 25, 50, and 75 %, respectively)

Normal hepatic lobes (hepatic lobes with no parasite and pathological lesion)

Abnormal hepatic lobes (hepatic lobes with *D. dendriticum* and pathological lesions)

### The relationship of oxidative stress markers with PCV and biochemical variables of liver injury

To investigate the relationship of oxidative stress markers with PCV and serum variables of liver injury, Pearson’s correlation test was performed on the paired data obtained from the individual parasitized cases. A significant positive correlation was seen between serum MDA concentration and the activity of AST (r=0.360, *P*=0.003). On the other hand, the concentration of serum MDA was inversely correlated with the values of PCV (r=−0.420, *P*=0.001). The concentration of MDA and FRAP in liver tissue revealed no significant correlation with the values of PCV and variables of liver injury (serum AST, GGT, bilirubin, and albumin).

### The relationship of oxidative stress markers with extent of liver injury

To investigate the relationship of oxidative stress markers with extent of liver injury in sheep with dicrocoeliasis, the number of hepatic lobes with pathological lesions was recorded by microscopic examination in each animal. The extent of pathological lesion was then classified as 1 (lesions in only one hepatic lobe), 2 (lesions in two lobes), 3 (lesions in 3 lobes) and 4 (lesions in all 4 lobes). Then, the relationship of oxidative stress markers with the pathological extent was evaluated by Spearman′s correlation test performed on the paired data obtained from the individual parasitized cases. There was no significant correlation between serum and liver MDA and FRAP concentrations and the extent of pathological lesions in the parasitized sheep.

### The relationship of oxidative stress markers with parasite burden

The number of *D. dendriticum* worms obtained per lamb varied from 10 to 18500 (1596.5±3211.7) in the parasitized animals. The relationship of serum and liver MDA and FRAP with the fluke’s number was evaluated by Spearman′s correlation test performed on the paired data obtained from the individual parasitized cases. No significant correlations were observed between serum and liver MDA and FRAP concentrations and the fluke’s number in the parasitized animals.

## Discussion

Oxidative stress and changes in antioxidants have been already implicated in the different parasitic diseases (14–17, 23–27). Although oxidative stress has been reported in sheep and rats suffering from dicrocoeliasis (12–13, 18), it is very important to evaluate the role of oxidative stress in the pathology of ovine dicrocoeliasis.

The results of the present study show that dicrocoeliasis in sheep also courses with oxidative stress as indicated by enhanced lipid peroxidation (liver and serum MDA) and anti-oxidant capacity (serum FRAP). This result was similar to those reported by others (12–13, 18, 28). High lipid peroxidation may be due to oxidation of molecular oxygen to produce superoxide radicals. This reaction is also the source of H_2_O_2_, which causes the production of MDA by initiating the peroxidation of unsaturated fatty acids in the membrane (29). The increased antioxidant capacity observed in the present study may be explained by up-regulation of antioxidants to counter the ROS (30). Indeed, the oxidants activate gene expression through antioxidant responsive elements (31).

Serum albumin is a negative acute phase protein and its synthesis in the liver decreases in hepatic infection and injury (32). In addition, because of its thiol groups, albumin is the most important extracellular antioxidant (33). In the present study, the concentration of serum albumin was decreased in the parasitized animals compared to the healthy animals, suggesting a reduction of the hepatic synthesis of this protein and/or consumption of this antioxidant component as free radical scavenger.

Host body needs an adequate supply of trace elements for the structure and function of some antioxidant enzymes that participate in the protection of cells against highly toxic ROS (e.g., zinc and copper for superoxide dismutase and iron for catalase (34–35). In the present study, decreased zinc and iron concentrations were found in the parasitized animals. Low serum trace elements have been reported in parasitic diseases (15–17, 36). Increased metabolism or consumption (37), inappetence, stress (38) or hyperthermia (39) could result in losses of trace elements during parasitic infections.

The level of PCV was significantly (*P*<0.05) lower in sheep with liver dicrocoeliasis than those of healthy controls. In addition, there was a significant negative correlation between MDA concentration and the level of PCV in sheep with dicrocoeliasis. The increased generation of ROS cause damage to biomolecules in which lipids are probably the most susceptible. Due to abundance of polyunsaturated fatty acids in the cell membrane and presence of iron, erythrocytes are highly susceptible to peroxidative damage (40). The significant negative correlation between MDA concentration and the level of PCV suggested enhanced oxidative damage to erythrocytes, either due to compromise in antioxidant defense or excess production of free radicals. These results agree with previously reported increased erythrocyte oxidation and destruction in sheep naturally infected with *F. hepatica* and *Trypanosoma evansi* (14; 41) and in sheep and camel infected with liver cystic echinococcosis (15–17). ROS could induce peroxidation of membrane lipids, impairment of enzymatic and structural proteins and inhibition of mitochondrial ATP synthesis. These changes are followed by the elevation in intracellular calcium, disruption of the cytoskeleton and the plasma membrane, and finally, cell death (42).

In the present study, the histological findings were similar to previous papers (13; 4), indicating that dicrocoeliasis had caused the hepatic damage. The pathological changes observed in dicrocoeliasis are caused by direct mechanical stimulation, probably due to the suckers of the adult flukes, fibrosis-promoting factors released by leukocytes and the toxic metabolites released by the adult flukes, inducing an inflammatory reaction (4). Oxidative stress may also have an important role in liver damage in experimentally induced dicrocoeliasis in hamsters (13, 18). Radicals produced during lipid peroxidation react with the SH group of cysteine, resulting in the formation of covalent bonds between lipids and membrane proteins. They also react with each other forming lipid–lipid covalent bonds. Subsequently, structural changes in cell membranes lead to increased membrane permeability. Therefore, cytosolic enzymes such as AST and ALT could release into the blood (7). In camel and sheep with liver cystic echinococcosis, a positive correlation was observed between serum MDA concentration and the values of hepatic markers (bilirubin and hepatic enzymes), suggesting that the enhanced lipid peroxidation may be linked to hepatic damage (16, 17). In the present study, no significant differences were observed for MDA and FRAP concentrations between normal and abnormal hepatic lobes in the parasitized animals. In addition, oxidative stress markers (serum and liver MDA and FRAP concentrations) showed no significant correlations with the extent of pathological lesions and variables of liver injury (serum GGT and bilirubin) in the parasitized sheep. The oxidative stress would not have an important role in the *D. dendriticum-*induced hepatic damage in sheep. Although increased MDA level was observed in the liver of sheep parasitized with *D. dendriticum*, the amount of lipid peroxidation was not as high as to induce hepatic damage. The biologic damage caused by free-radical-mediated mechanisms might be prevented by antioxidants inducible by oxidative stress (43). The significant positive correlation between serum MDA concentration and the activity of AST in the parasitized animals might be attributed to the enhanced oxidative damage to erythrocytes, not to the hepatic damage. The significant negative correlation between serum MDA concentration and the level of PCV supports such a hypothesis. The AST is found in erythrocytes, and the increased destruction of erythrocytes will result to elevated serum AST activity (44).

There is a tendency for an increase in oxidative stress and lipid peroxidation by increasing the worm burden in human with schistosomiasis. The ROS are produced near parasites in the liver and they are responsible for destroying the parasites (45). In the present study, no clear relationship was observed between the oxidative stress markers (serum and liver MDA and FRAP concentrations) and the fluke’s number in the liver of parasitized animals. The oxidative stress observed in the sheep parasitized with *D. dendriticum*, is a systemic response to the parasite and its severity is independent of parasite burden. Systemic inflammation induced by various pathogens is associated with an increase in ROS generation (46).

## Conclusion

Oxidative stress may play an important role in the erythrocyte destruction in sheep naturally infected with *D. dendriticum.* However, no evidence was observed to substantiate that oxidative stress induce hepatic damage in ovine dicrocoeliasis. In addition, no clear relationship was observed between the oxidative stress and parasite burden. Dicrocoeliasis in sheep is associated with decreased zinc and iron concentrations.
